# Validation of the Lithuanian Version of the International Restless Legs Syndrome Study Group Rating Scale for Restless Legs Syndrome

**DOI:** 10.3390/medicina61061028

**Published:** 2025-05-31

**Authors:** Domantė Lipskytė, Tadas Vanagas, Evelina Pajėdienė

**Affiliations:** 1 Faculty of Medicine, Medical Academy, Lithuanian University of Health Sciences, 44307 Kaunas, Lithuania; 2 Department of Neurology, Medical Academy, Lithuanian University of Health Sciences, 44307 Kaunas, Lithuania; tadas.vanagas@lsmu.lt (T.V.); evelina.pajediene@lsmu.lt (E.P.); 3 Department of Neurology, Kauno Klinikos, Hospital of Lithuanian University of Health Sciences, 50161 Kaunas, Lithuania

**Keywords:** sleep disorders, restless legs syndrome, international restless legs syndrome rating scale, validation, screening tool, periodic limb movement disorder

## Abstract

*Background and Objectives*: According to the literature, Restless Legs Syndrome (RLS) often remains underdiagnosed, with only a small proportion of individuals experiencing symptoms receiving an official diagnosis, highlighting the need for effective screening and diagnostic tools. The International Restless Legs Syndrome Study Group Rating Scale (IRLS) is a widely used tool for assessing the severity of Restless Legs Syndrome (RLS). However, a validated Lithuanian version has not yet been established. This study aimed to validate the Lithuanian version of the IRLS and assess its reliability, diagnostic performance, and correlation with clinical and demographic factors. *Materials and Methods*: This retrospective study included 136 patients who completed the Lithuanian version of the IRLS and underwent polysomnographic and clinical evaluations at the Department of Neurology of the Lithuanian University of Health Sciences between 2018 and 2024. A total of 134 patients were analyzed: 66 with clinically confirmed RLS and 68 controls without sleep disorders. Statistical analysis included the Mann–Whitney U test, chi-squared tests, Receiver Operating Characteristics (ROC) curve analysis, multivariate logistic regression, and Akaike Information Criterion (AIC). *Results*: The Lithuanian IRLS demonstrated good diagnostic accuracy with an Area Under the Curve (AUC) value of 0.843 (95% CI: 0.782–0.904), with an optimal cut-off score of 7.50, resulting in high sensitivity (92.4%) and moderate specificity (66.2%). Multivariate regression identified higher IRLS scores (OR = 1.212, 95% CI: 1.084–1.356, *p* < 0.001) and a higher periodic limb movements of sleep arousal index (PLMSAI) (OR = 1.961, 95% CI: 1.036–3.712, *p* = 0.039) as significant independent predictors of RLS. After adjustments for age and sex, both IRLS scores and PLMSAI remained statistically significant predictors. *Conclusions*: the Lithuanian version of IRLS is a valid and reliable instrument for assessing RLS severity. Its diagnostic performance supports its use in clinical and research settings for identifying and monitoring RLS in Lithuanian population.

## 1. Introduction

Restless Leg Syndrome (RLS) is a prevalent sensorimotor condition, affecting approximately 7.12% of the population, characterized by an irresistible urge to move legs that arises or worsens during periods of rest, typically in the evening or at night, and is relieved or resolved through movement [1,2]. The manifestation of RLS symptoms is shaped by a combination of genetic factors, environmental factors, and associated comorbidities, such as iron deficiency, kidney disease, cardiovascular disorders, diabetes, and various neurological, rheumatological, and respiratory conditions [1,3]. This prevalent syndrome significantly impacts sleep, mental health, and quality of life. A study analyzing the influence of RLS on daily life showed that patients with RLS rated their vitality, bodily pain, mental health, and social functioning significantly lower than the general population [4]. Additionally, another study suggests that RLS is positively associated with excessive daytime sleepiness, which further impairs concentration and potentially contributes to learning difficulties [5].

RLS is diagnosed based on the clinical criteria established by the International Restless Legs Syndrome Study Group (IRLSSG), requiring an urge to move the legs with associated discomfort, symptoms worsen during rest, improve with movement, and are more pronounced in the evening or night, provided these symptoms are not explained by other conditions [6]. The diagnostic criteria for RLS outlined in the International Classification of Sleep Disorders, Third Edition (ICSD-3), align closely with those established by IRLSSG, with the added stipulation that symptoms must lead to concern, distress, sleep distribution, or functional impairment [7]. In RLS diagnosis, polysomnography (PSG) is not required due to its limited diagnostic value [8]. However, it may be indicated in cases of diagnostic ambiguity, suspecting periodic limb movements of sleep (PLMS) contributing to sleep disruption, or when comorbid sleep disorders, such as obstructive sleep apnea (OSA), are suspected [9,10].

As previously noted, RLS significantly impairs quality of life by contributing to reduced sleep efficiency, chronic insomnia, and an increased prevalence of depressive symptoms [11]. Interestingly, a Finnish publication revealed that despite 81% of patients with RLS symptoms seeking medical attention, only 6.2% received an official diagnosis, highlighting a gap in the recognition of the disorder. This emphasizes the need for improved diagnostic tools and better awareness among healthcare providers [12].

A variety of rating scales have been developed to streamline the diagnosis of RLS in larger populations while assessing the overall severity of the condition [13,14]. The International Restless Legs Syndrome Study Group Rating Scale (IRLS) was published in 2003 and it is widely used in RLS research and clinical practice, serving as the primary instrument to determine RLS severity in therapeutic studies. It showed sensitivity of 82% and specificity of 92% [13]. The scale enhances diagnostic accuracy and treatment effectiveness by providing objective measures of symptom severity, thereby improving patient care in RLS management [15]. The IRLS has demonstrated solid reliability and validity in diagnosing RLS, with studies supporting its ability to accurately measure RLS severity across diverse patient populations and clinical settings [16].

The absence of a validated Lithuanian version of the IRLS limits the ability to accurately diagnose and assess treatment outcomes for RLS in Lithuania, hindering effective clinical management. Without a culturally adapted and validated tool, there is an increased risk of misdiagnosis and suboptimal monitoring of treatment response, impacting the quality of patient care.

The objective of this study was to evaluate Lithuanian version of IRLS sensitivity and specificity within the Lithuanian population. By addressing this diagnostic gap, this study aims to assess the reliability and utility of the Lithuanian version of the IRLS in facilitating an accurate diagnosis of RLS.

## 2. Materials and Methods

### 2.1. Study Design and Participants

To conduct this study, approval was obtained from the Kaunas Regional Biomedical Research Ethics Committee on 30 January 2025 (Approval No. BE-2-7). This retrospective study included 136 adult patients who were selected from clinical records based on referral for routine video polysomnography (PSG) at the Department of Neurology, Hospital of Lithuanian University of Health Sciences. Participants were referred for PSG between 2018 and 2024 due to subjective sleep disturbances identified during routine neurological consultations in the outpatient department. For each participant, responses to the Lithuanian version of sleep-related questionnaires (Epworth Sleepiness Scale (ESS), Insomnia Severity Index (ISI), and International Restless Legs Syndrome Study Group Rating Scale (IRLS), Berlin Questionnaire) were collected. Additionally, comprehensive medical records were reviewed, including PSG-driven parameters, presence of comorbid conditions, and ongoing medication use. To ensure diagnostic accuracy, final diagnoses were cross-referenced with clinical records and physician assessments.

### 2.2. Formation of Groups

**Figure 1:**
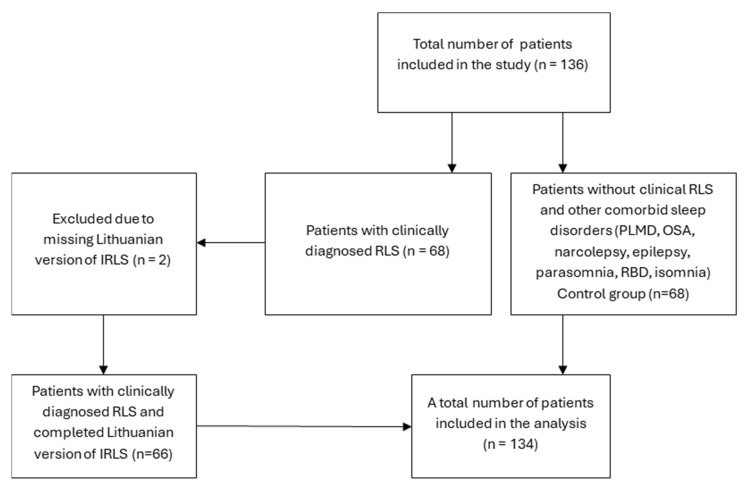
Flow chart of studied patients.

Patients who completed sleep questionnaires, underwent all-night video PSG, and had available clinical data, were included. The RLS group consisted of patients with a clinically confirmed RLS diagnosis. The control group consisted of patients who had no clinical diagnosis of sleep disorders, including RLS, Periodic Limb Movement Disorder (PLMD), obstructive sleep apnea (OSA), narcolepsy, epilepsy, parasomnia, REM sleep behavior disorder (RBD), and insomnia. Patients with incomplete sleep questionnaires or missing medical records were also excluded. The formation of groups is illustrated in Figure 1.

Restless Legs Syndrome (RLS), Periodic Limb Movement Disorder (PLMD), obstructive sleep apnea (OSA), REM sleep behavior disorder (RBD), International Restless Legs Syndrome Study Group Rating Scale (IRLS) were used to assess patients.

### 2.3. International Restless Legs Syndrome Scale

The International Restless Legs Syndrome Study Group Rating Scale (IRLS) was translated into Lithuanian by a clinical neurologist to ensure linguistic and conceptual accuracy. The scale consisted of ten items designed to assess key aspects of RLS, including the presence and severity of limb discomfort, the urge to move the legs, symptom relief with movement, the impact on sleep, daytime fatigue or somnolence, overall symptom severity, symptom frequency, average symptom burden, the extent to which RLS interferes with daily activities, and associated mood disturbances. Each item was scored on a five-point ordinal scale, ranging from 0 (none) to 4 (very severe), with a maximum total score of 50. RLS severity was classified as follows: 0 points—no RLS, 1–10 points—mild RLS, 11–20 points—moderate RLS, 21–30 points—severe RLS, and 31–40 points—very severe RLS.

### 2.4. Statistical Analysis

Statistical analysis was performed using IBM SPSS Statistics 30.0 (IBM Corporation, New York, NY, USA). Descriptive statistics were presented as absolute and percentage values, as well as mean ± standard deviation (SD) for the continuous variables. Group comparisons were conducted using the Mann–Whitney U test for non-normally distributed data. The chi-squared (χ^2^) test was applied to evaluate associations between categorical variables. To assess the diagnostic accuracy of the RLS questionnaire, Receiver Operating Characteristics (ROC) curve analysis was performed. The results are expressed using Area Under the Curve (AUC); 95% confidence intervals (CIs) are reported. Additionally, multivariate logistic regression was performed to evaluate predictors of RLS. Akaike Information Criterion (AIC) was calculated. A *p*-value of <0.05 was considered statistically significant for all the tests.

## 3. Results

### 3.1. Characteristics of Patient Groups

A total of 134 patients were included in the analysis, consisting of 66 patients with a clinical diagnosis of RLS and 68 patients in the control group. Among participants diagnosed with RLS, 41 were female and 27 were males, whereas the control group included 39 females and 29 males. No significant difference was found in gender distribution between groups (*p* = 0.727). A significant age difference was observed between RLS and control groups (respectively, 55.34 ± 16.68 (95% CI: 51.07–59.62) vs. 36.13 ± 11.80 (95% CI: 33.28–38.99) years, *p* < 0.001). The mean body mass index (BMI) was significantly higher in the RLS group compared to the controls (28.09 ± 4.65; 95% CI: 26.9–29.28 vs. 24.24 ± 3.67, 95% CI: 23.35–25.13, *p* < 0.001).

ESS scores did not significantly differ between the groups (*p* = 0.989), while the mean IRLS scores were significantly higher in the RLS group (19.8 ± 9.14; 95% CI: 17.04–21.72) compared to the controls (6.96 ± 8.68; 95% CI: 4.85–9.06) (*p* < 0.001), demonstrating its ability to differentiate patients with and without RLS. Demographic and clinical characteristics are presented in Table 1.

Additionally, associations between RLS and common comorbidities, including primary arterial hypertension (PAH), diabetes, atrial fibrillation (AF), heart failure (HF), and multiple sclerosis (MS), were tested. Significant associations were found between RLS and PAH (χ^2^ = 12.12, *p* < 0.001), RLS and diabetes (χ^2^ = 5.78, *p* = 0.016), and RLS and HF (χ^2^ = 7.46, *p* = 0.006). No significant associations were observed with AF (*p* = 0.402) or MS (*p* = 0.315). Prevalence of comorbidities is presented in Table 2.

A statistically significant difference in BMI was found between patients in the RLS group at a high and low risk of OSA, based on the Berlin Questionnaire. The high-risk group demonstrated higher BMI values (t(63) = −2.25, *p* = 0.028, 95% CI: −4.76 to −0.29). The effect size was moderate (Cohen’s d = 0.558). Additionally, a statistically significant positive correlation was observed between BMI and the apnea–hypopnea index (AHI) in the RLS group (ρ = 0.402, *p* < 0.001).

### 3.2. International Restless Legs Syndrome Study Group Rating Scale Score Correlations

A significant difference was confirmed in the IRLS scores between the groups (U = 706.50, Z = −6.884, *p* < 0.001). However, no significant difference was observed in ESS scores between groups (*p* = 0.989).

In the RLS group, a significant positive correlation was identified between the IRLS scores and ISI (r = 0.384, *p* = 0.002), demonstrating that greater RLS severity is associated with more pronounced subjective sleep disturbances. No significant correlations were observed in the RLS group between RLS scores and PAH (U = 276, Z = −1.496, *p* = 0.135), AF (U = 71.50, Z = −0989, *p* = 0.322), or HF (U = 136, Z = −0.809, *p* = 0.419). Additionally, no notable correlations were found between IRLS scores and diabetes (U = 155, Z = −0.789, *p* = 0.430).

No significant correlations were observed between IRLS scores and periodic limb movements of sleep (PLMS) (ρ = −0.222, *p* = 0.073) or the PLMS sleep arousals index (PLMSAI) (ρ = −0.196, *p* = 0.134). Correlations between IRLS scores and clinical variables are presented in Table 3.

### 3.3. Validation of the Lithuanian Version of the International Restless Legs Syndrome Study Group Rating Scale

**Figure 2:**
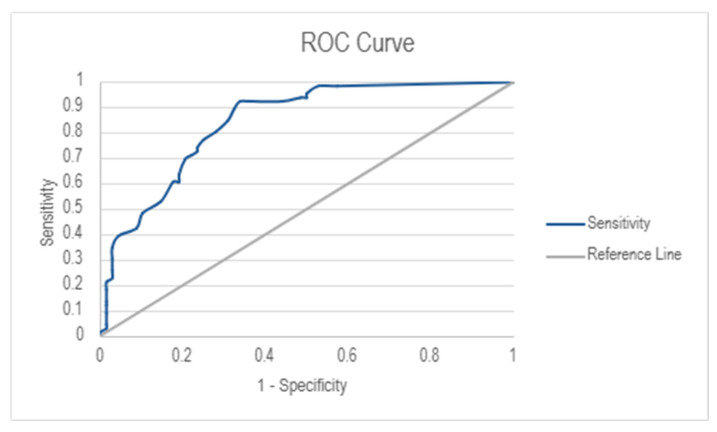
ROC analysis: diagnostic performance of IRLS.

The diagnostic performance of the IRLS was assessed using Receiver Operating Characteristics (ROC) analysis (Figure 2). The Area Under the Curve (AUC) was 0.843 (95% Cl: 0.782–0.904), indicating good discriminative ability. The optimal cut-off score for RLS diagnosis was determined to be 7.50, resulting in a high sensitivity score of 92.4% and a moderate specificity score of 66.2%.

**Figure 3:**
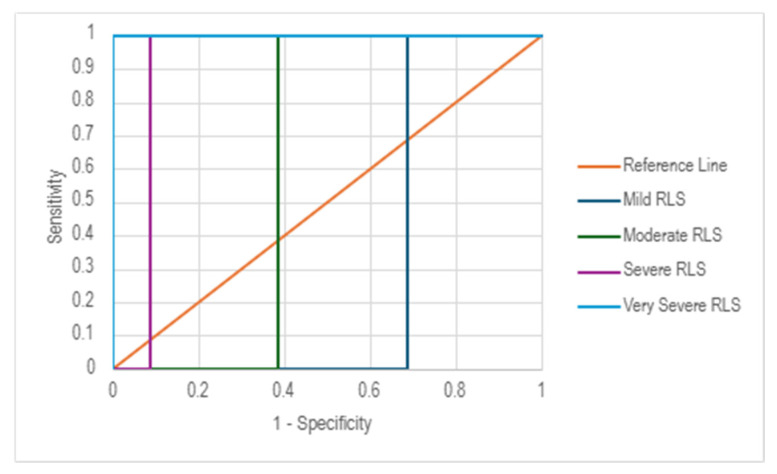
ROC analysis: diagnostic performance across different RLS severity levels.

The ROC curve analysis for the Lithuanian version of the IRLS demonstrated varying levels of diagnostic accuracy across different severity levels of RLS. As shown in the ROC plot (Figure 3), the AUC values increased with RLS severity, indicating stronger discriminative ability for severe and very severe RLS cases. The AUC values were 0.314 (95% CI: 0.225–0.403) for mild RLS, 0.614 (95% CI: 0.519–0.709) for moderate RLS, 0.913 (95% CI: 0.859–0.967) for severe RLS, and 1.000 (95% CI: 1.000–1.000) for very severe RLS, showing an increasing trend in classification accuracy with higher RLS severity. The cut-off values obtained for each severity level were 1.50 for mild RLS, 10.50 for moderate RLS, 20.50 for severe RLS, and 30.50 for very severe RLS. The ROC curves for severe and very severe RLS categories aligned closely with the *y*-axis, demonstrating a great classification performance, whereas mild and moderate RLS classifications exhibited lower specificity and sensitivity.

To determine the predictors of RLS diagnosis, a multivariate logistic regression model was created. The overall model was statistically significant (χ^2^ = 54.32, df = 5, *p* < 0.001) with a Nagelkerke R^2^ value of 0.726, indicating that the model explains 72.6% of the variance in RLS diagnosis. The IRLS scores were the strongest predictor of RLS (OR = 1.212, 95% CI: 1.084–1.356, *p* < 0.001), confirming its diagnostic utility. A trend was observed that a higher BMI was associated with increased RLS risk (OR = 1.316, 95% CI: 0.992–1.745, *p* = 0.057). The PLMSAI was also a significant independent predictor (OR = 1.961, 95% CI: 1.036–3.712, *p* = 0.039). PAH and PLMS were not statistically significant. Model fit was tested by the Hosmer–Lemeshow test (χ^2^ = 11.08, df = 8, *p* = 0.197), and the Akaike Information Criterion (AIC) value was 51.87. After adjusting for age and sex, IRLS scores remained a strong predictor of RLS (adjusted OR = 1.187, 95% CI: 1.054–1.337, *p* = 0.005), and PLMSAI also remained statistically significant (adjusted OR = 1.968, 95% CI: 1.019–3.801, *p* = 0.044). No other variables reached significance after adjustment. The adjusted model showed a slightly higher AIC value of 53.87. The results are illustrated in Table 4.

## 4. Discussion

This study aimed to assess the diagnostic accuracy of the Lithuanian version of the IRLS, with a particular focus on its sensitivity and specificity. The results indicate a strong diagnostic performance, with a sensitivity of 92.4% and a specificity of 66.2%. AUC was 0.843 (95% CI: 0.782–0.904), demonstrating good discriminatory capability. Notably, AUC values increased with the severity of RLS, suggesting the scale is particularly effective in detecting severe and very severe cases, whereas sensitivity and specificity were lower in cases with mild and moderate symptoms, indicating that additional clinical evaluations may be necessary in the early stages of RLS.

The IRLS has undergone validation in various linguistic and cultural contexts, consistently demonstrating high reliability. For example, the Japanese version showed great reliability, with an intra-class correlation coefficient (ICC) of 0.877 (95% CI: 0.802–0.925) [17]. Similarly, the Brazilian Portuguese version of the IRLS demonstrated robust reliability, with Cronbach’s alpha values reaching approximately 0.80, further supporting its applicability in diverse populations [18]. In our study, IRLS scores accounted for 72.6% of the variance in RLS diagnosis, consistent with the prior findings that identified a dominant severity factor explaining over 59% of item variance [15]. Furthermore, a meta-analysis across 14 studies reported median sensitivity and specificity values of 88% and 90%, respectively, for various RLS diagnostic tools, underscoring their overall high diagnostic accuracy [19].

In our study, the Lithuanian version of the IRLS demonstrated strong validity, with IRLS scores significantly predicting RLS diagnosis even after adjustments for age and sex. This finding reflects previous validations showing the IRLS scale to be a reliable tool across different populations [15]. No significant correlations were observed between IRLS scores and either the PLMS or PLMSAI, suggesting that subjective symptom severity does not necessarily reflect objective motor activity during sleep, consistent with the previous findings [20]. Despite the lack of correlation, PLMSAI remained an independent predictor of RLS after adjustment, supporting the idea that sleep-related arousals linked to limb movements contribute to disease severity [21]. Additionally, the slight increase in the AIC after adjustment reflects the added complexity of the model when accounting for demographic covariates, such as age and sex, maintaining strong model precision. Overall, these results reinforce the Lithuanian IRLS questionnaire as a reliable tool and emphasize the importance of integrating both subjective assessments and objective sleep-related measures when evaluating RLS severity.

A significant correlation was observed between IRLS and ISI scores (*p* = 0.002), supporting the association between RLS severity and insomnia symptoms. Similar findings have been reported previously, including a study assessing symptom progression before and after the COVID-19 lockdown, which found worsening RLS symptoms were accompanied by increased insomnia severity [22]. Another study on dialysis patients reported insomnia in 35% of those with RLS, compared to 16% in control subjects [23].

In contrast, our study found no significant difference in ESS scores between RLS patients and controls, suggesting that excessive daytime sleepiness may not be a consistent feature distinguishing RLS. However, other investigations have reported higher ESS scores in RLS populations. A large-scale study involving 1497 participants found significantly elevated ESS scores in individuals with RLS symptoms compared to controls (10.22 ± 6.32 vs. 9.01 ± 5.95, *p* = 0.002) [11]. Similarly, another study noted that RLS patients had higher ESS scores (8.5 ± 4.9) than controls (7.3 ± 4.2), with 57% of RLS participants scoring above 10, indicating clinically significant daytime sleepiness [24]. These data suggest that the lack of a statistically significant association in our findings may be attributed to the relatively small sample size.

Our analysis also revealed that patients with RLS were significantly older than control participants (55.34 vs. 36.13 years, *p* < 0.001) and had a higher BMI (28.09 vs. 24.24, *p* < 0.001). These results are supported by the previous research indicating that RLS prevalence increases with age: 3% of individuals aged 18–29 years, 10% of those aged 30–79 years, and 19% of individuals aged 80 years and older reported RLS symptoms occurring at least five nights per month. In addition, RLS has been associated with elevated BMI, smoking, physical inactivity, and comorbidities, such as diabetes [25]. Consistent with these findings, our study identified a higher prevalence of PAH, diabetes, and HF among RLS patients. However, no significant associations were observed between IRLS scores and these comorbidities, suggesting that while such conditions may be more frequent in individuals with RLS, they do not directly influence symptom severity as measured by the IRLS.

In terms of BMI, our findings indicate a potential role of elevated body weight in increasing the risk of OSA among RLS patients. Individuals categorized as high-risk for OSA based on the Berlin Questionnaire had significantly higher BMI values than those considered as low risk (*p* = 0.028). Additionally, BMI showed a positive correlation with the AHI (*p* < 0.001), suggesting that excess weight may contribute to OSA severity in patients with RLS. These findings are consistent with prior studies reporting an association between obesity and OSA in both the general population and among individuals with RLS. For instance, previous reports noted that obesity may increase the risk of OSA in RLS patients due to shared mechanisms, such as upper airway obstruction and systemic inflammation. In this study, 70.4% of patients with RLS were found to be overweight or obese, and OSA was diagnosed, on average, 21 months following the initial RLS diagnosis, highlighting the frequent co-occurrence and potential delayed recognition of OSA in patients with RLS [26]. Other studies also emphasize the importance of OSA screening in RLS patients, particularly given overlapping clinical features and common risk factors, like elevated BMI [27]. These observations highlight the importance of monitoring BMI and evaluating sleep-disordered breathing in individuals with RLS.

Multiple mechanisms may explain the relationship between obesity and RLS. Vascular dysfunction has been implicated in both conditions [28], while dopaminergic system abnormalities—particularly reduced dopamine D2 receptor availability—may play a role in both obesity and RLS [29]. Furthermore, a sedentary lifestyle and metabolic dysregulation may exacerbate RLS symptoms, particularly in patients with increased daytime sleepiness and reduced physical activity levels [30].

In this study, several limitations should be acknowledged. First, the study sample was derived from a specialized sleep clinic, potentially limiting the generalizability of findings to broader or primary care populations. This may have resulted in an overrepresentation of more severe or complex cases. Psychometric properties, such as test–retest reliability and internal consistency (e.g., Cronbach’s alpha), were not assessed, representing a methodological limitation in the validation process. Future research should aim to address these aspects to strengthen the reliability of the IRLS. Second, differences in age and BMI between groups may have affected the objectivity of the group selection. Although adjusted in the analysis, better control through matching or stratification would have improved group comparability. Third, some predictors showed wide confidence intervals, indicating limited precision likely due to the sample size. Although strict translation procedures were employed, subtle cultural and linguistic differences specific to the Lithuanian context may have influenced patient responses. Another important consideration is the potential adaptation of the IRLS for digital platforms or telehealth use to enhance both its acceptability and clinical utility. Lastly, aspects such as patient-reported acceptability, cost-effectiveness, and practical utility of the Lithuanian IRLS in primary care remain unassessed and could be a potential future research implication.

## 5. Conclusions

Our study findings confirm the clinical utility of the Lithuanian version of the IRLS in identifying symptoms of RLS. The tool demonstrated high sensitivity and moderate specificity, making it a valuable instrument for detecting RLS in clinical practice. However, its diagnostic accuracy was reduced in cases of mild or moderate symptom severity, indicating that additional clinical assessments may be necessary in early-stage presentations.

The IRLS scores were identified as the strongest independent predictor of RLS, with the PLMSAI also contributing significantly to diagnostic accuracy. Additionally, the association observed between higher BMI, increased risk of OSA, and greater AHI values highlights the need to consider weight-related factors in the management of patients with RLS. These findings support previous evidence suggesting a shared pathophysiological basis between RLS, obesity, and sleep-disordered breathing.

Taken together, these findings support the use of a more targeted screening approach that integrates validated questionnaires with key clinical indicators. This strategy may improve the early identification of RLS and comorbid conditions, particularly in outpatient and primary care settings, where timely recognition and intervention are critical to improving patient outcomes.

## Figures and Tables

**Table 1:** Comparison of demographic and clinical characteristics between RLS and control groups (data presented in mean ± SD).

Variable	RLS Group (*n* = 66)	Control Group (*n* = 68)	*p*-Value
Age (years)	55.34 ± 16.68	36.13 ± 11.80	<0.001
Gender (Female/Male)	40 (60.6%)/26 (39.4%)	40 (58.8%)/28 (41.2%)	0.727
BMI (kg/m^2^)	28.09 ± 4.65	24.24 ± 3.67	<0.001
ESS Score	7.62 ± 5.10	7.59 ± 4.84	0.989
IRLS Score	19.8 ± 9.14	6.96 ± 8.68	<0.001

BMI—body mass index, ESS—Epworth Sleep Scale, and IRLS—International Restless Legs Syndrome Study Group Rating Scale.

**Table 2:** Prevalence of comorbidities in RLS and control groups.

Comorbidity	RLS Group (*n* = 66)	Control Group (*n* = 68)	Chi square, χ^2^	*p*-Value
PAH	22 (33.3%)	6 (8.8%)	12.12	<0.001
Diabetes	8 (12.1%)	1 (1.5%)	5.78	0.016
HF	7 (10.6%)	0 (0.0%)	7.46	0.006
AF	4 (6.1%)	2 (2.9%)	0.70	0.402
MS	0 (0.0%)	1 (1.5%)	1.00	0.315

PAH—primary arterial hypertension, HF—heart failure, AF—atrial fibrillation, and MS—multiple sclerosis.

**Table 3:** Correlation between IRLS scores and clinical variables.

Variable	Test Value (ρ/U and Z)	*p*-Value
ISI	ρ = 0.384	0.002
PAH	U = 276, Z = −1.496	0.135
AF	U = 71.50, Z = −0.989	0.322
HF	U = 136, Z = −0.809	0.419
Diabetes	U = 155, Z = −0.789	0.430
PLMS	ρ = −0.222	0.073
PLMSAI	ρ = −0.196	0.134

ISI—Insomnia Severity Index, PAH—primary arterial hypertension, AF—atrial fibrillation, HF—heart failure, PLMS—periodic limb movements of sleep, PLMSAI—periodic limb movement of sleep arousals index.

**Table 4:** Multivariate logistic regression analysis of predictors for RLS diagnosis.

Predictor	Multivariate Analysis			Multivariate Analysis—Adjusted for Age and Sex		
	OR (95% CI)	*p*-Value	AIC	OR (95% CI)	*p*-Value	AIC
IRLS Scores	1.212 (1.084–1.356)	<0.001	51.87	1.187 (1.054–1.337)	0.005	53.87
PLMSAI	1.961 (1.036–3.712)	0.039	51.87	1.968 (1.019–3.801)	0.044	53.87
PLMS	0.965 (0.885–1.052)	0.419	51.87	0.960 (0.877–1.051)	0.378	53.87
BMI (kg/m^2^)	1.316 (0.992–1.745)	0.057	51.87	1.345 (0.989–1.831)	0.059	53.87
PAH	0.734 (0.089–6.082)	0.774	51.87	0.420 (0.038–4.580)	0.476	53.87

IRLS—International Restless Legs Syndrome Study Group Rating Scale, BMI—body mass index, PAH—primary arterial hypertension, PLMS—periodic limb movements of sleep, PLMSAI—periodic limb movements of sleep arousal index, AIC—Akaike Information Criterion.

## Data Availability

The original contributions presented in this study are included in the article. Further inquiries can be directed to the corresponding authors.
